# Spatially-resolved intracellular sensing of hydrogen peroxide in living cells

**DOI:** 10.1038/srep16929

**Published:** 2015-11-20

**Authors:** Emilie A. K. Warren, Tatiana S. Netterfield, Saheli Sarkar, Melissa L. Kemp, Christine K. Payne

**Affiliations:** 1 School of Chemistry and Biochemistry, Georgia Institute of Technology, Atlanta, GA 30332, USA; 2 The Wallace H. Coulter Department of Biomedical Engineering, Georgia Institute of Technology and Emory University, Atlanta, GA 30332, USA; 3 Parker H. Petit Institute for Bioengineering and Biosciences, Georgia Institute of Technology, Atlanta, GA 30332, USA

## Abstract

Understanding intracellular redox chemistry requires new tools for the site-specific visualization of intracellular oxidation. We have developed a spatially-resolved intracellular sensor of hydrogen peroxide, HyPer-Tau, for time-resolved imaging in live cells. This sensor consists of a hydrogen peroxide-sensing protein tethered to microtubules. We demonstrate the use of the HyPer-Tau sensor for three applications; dose-dependent response of human cells to exogenous hydrogen peroxide, a model immune response of mouse macrophages to stimulation by bacterial toxin, and a spatially-resolved response to localized delivery of hydrogen peroxide. These results demonstrate that HyPer-Tau can be used as an effective tool for tracking changes in spatially localized intracellular hydrogen peroxide and for future applications in redox signaling.

Hydrogen peroxide (H_2_O_2_) is an essential extracellular and intracellular signaling molecule that reacts with protein cysteine thiols to confer reversible post-translational modifications^1^,^2^,^3^,^4^. Kinetic analyses of thiol disulfide systems using *in vitro*-determined rate constants suggest that many well-established protein thiol targets in the cellular milieu, even with low thiol pKas, are likely not competitive for two-electron exchange due to the abundance of other reducing molecules^5^,^6^. Consequently, there is great interest in investigating other means by which intracellular H_2_O_2_ results in protein oxidation for redox signaling. One such mechanism is the existence of microdomains of subcellular H_2_O_2_ production in close proximity to desired protein thiol targets^7^,^8^. Spatial characterization of H_2_O_2_ within live cells is imperative to understanding these dynamic biochemical events; however, technical limitations abound. While intracellular small molecule probes of reactive oxygen species^9^, including H_2_O_2_^10^,^11^,^12^,^13^,^14^,^15^, exist, they lack either specificity (the ability to distinguish between singlet oxygen, superoxide, hydroxyl radicals, and peroxides)^16^ or spatial resolution, instead functioning as diffuse cytosolic or organellar sensors. Thus, development of protein-based redox sensors has provided a significant advance in the fundamental understanding of the role of H_2_O_2_ in intracellular signaling and cell-cell communication^17^,^18^,^19^,^20^,^21^. Here, we develop a new ratiometric fusion protein sensor, HyPer-Tau, for spatially resolving intracellular and extracellular H_2_O_2_ gradients by tethering to a microtubule-binding protein, Tau.

## Results and Discussion

### Cellular localization of HyPer

**Figure 1:**
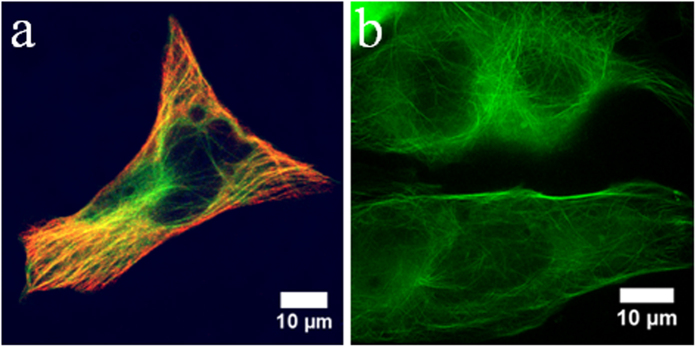
HyPer-Tau is a microtubule-localized sensor of intracellular H_2_O_2_. (**a**) Fluorescence microscopy image of a fixed and permeabilized HeLa cell showing colocalization of HyPer-Tau (green) with an antibody against tubulin (red). Individual images used to construct this two-color image are included in Supplementary Figure 2. (**b**) Super-resolution fluorescence microscopy image of live HeLa cells shows HyPer-Tau localized to the microtubules.

HyPer consists of yellow fluorescent protein (YFP) inserted into a bacterial hydrogen peroxide-sensing protein (OxyR)^22^ developed by Belousov *et al.* for the detection of H_2_O_2_ in cells^18^. When HyPer is oxidized, the excitation maximum shifts from 420 nm to 500 nm. The emission maximum remains at 516 nm. Plasmids are currently commercially available from Evrogen (Moscow, Russia) for expression of HyPer in the nucleus, cytosol, or mitochondria. Academic researchers have developed plasma membrane and ER-localized versions^23^, as well as PDGF, EDGF, and PIP3 fusions^21^,^24^. Expression of HyPer at these domains provides subcellular information, but lacks the spatial resolution necessary for comprehensive intracellular mapping of H_2_O_2_. Anchoring HyPer to the microtubules limits diffusion of the protein in the cytosol and provides an intracellular “grid” to map H_2_O_2_. Tau is a small (440aa) microtubule-binding protein that binds tightly to tubulin (1.1 μM binding affinity)^25^. The HyPer-Tau construct (Supplementary Figure 1) was generated using standard molecular biology methods, described in Materials and Methods. Expression of Hyper-Tau in HeLa cells shows localization with microtubules using both immunofluorescence (Fig. 1a and Supplementary Figure 2) and live cell super-resolution fluorescence microscopy (Fig. 1b).

### Intracellular response to extracellular H_2_O_2_

**Figure 2:**
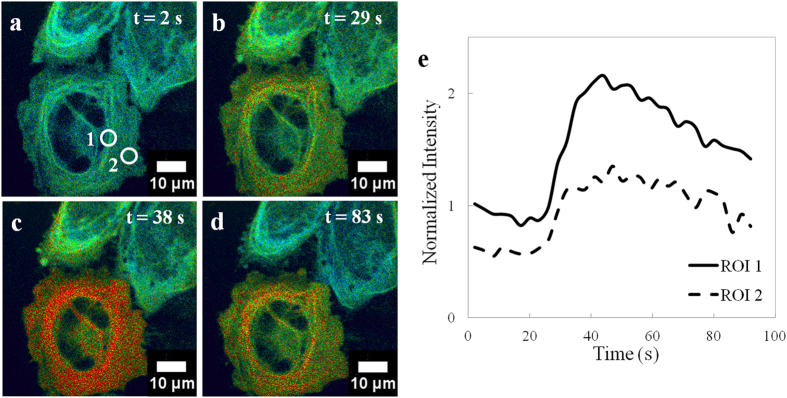
Effect of H_2_O_2_ on HyPer-Tau expressing HeLa cells. (**a–d**) H_2_O_2_ (100 μM) was added to cells and images were recorded at a rate of 1 Hz with a spinning disk confocal microscope. The pseudocolor images (0–256 scaling, red is the greatest change) represent the ratio of emission at 516 nm obtained from excitation at 488 nm versus 405 nm. (**e**) Two regions of interest (ROIs, white circles) were selected and the emission ratio in these regions was plotted as a function of time.

To first probe the intracellular response to exogenous H_2_O_2_, HeLa cells were transfected with HyPer-Tau and imaged with a spinning disk fluorescence microscope immediately following the addition of H_2_O_2_ (100 μM). Imaging multiple cells simultaneously shows a cell-specific response, as well as intracellular variations in oxidation (Fig. 2). Of three cells in the field of view, one shows strong oxidation while the other two cells show a minimal response (Fig. 2a–d). Within a single cell, the response also varies (Fig. 2e), illustrating the heterogeneity of intracellular oxidation. The response of HyPer-Tau to H_2_O_2_ was dose-dependent (Supplementary Figure 3).

### Intracellular response to intracellular H_2_O_2_

**Figure 3:**
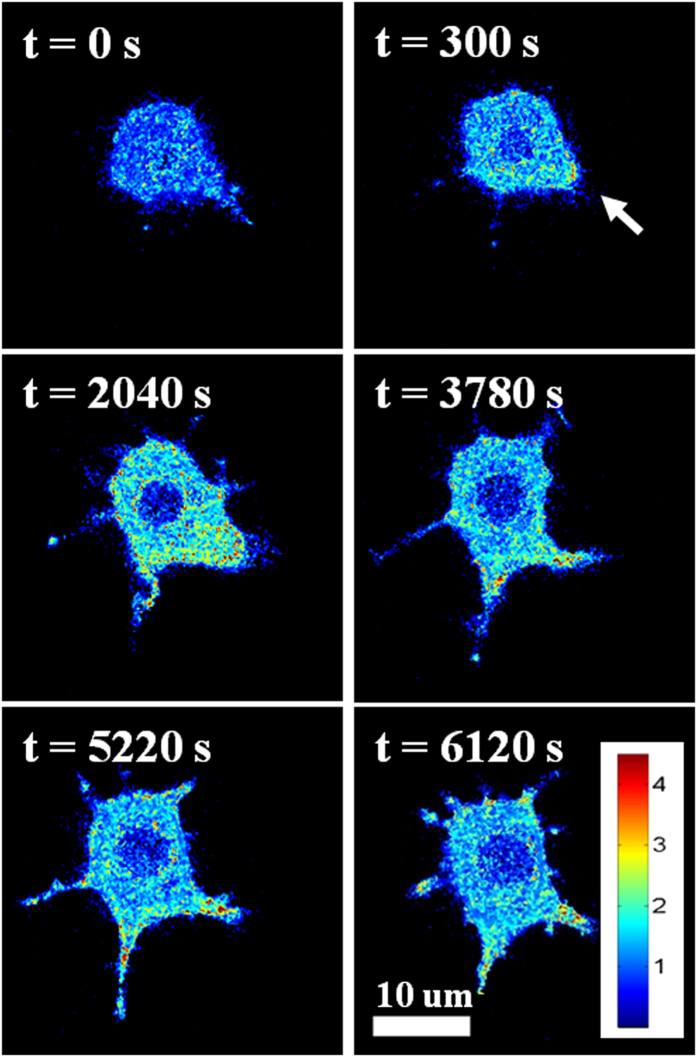
Intracellular sensing of endogenously-generated H_2_O_2_. Macrophage cells (RAW 264.7) imaged with a spinning disk confocal microscope following stimulation with 14.3 μg/mL LPS. The white arrow highlights a site of filopodia formation. For the complete time series, see the movie provided in Supplementary Movie 1. These images are representative of 3 cells from 2 experiments. A negative control is shown in Supplementary Figure 4.

Figure 2 shows the intracellular response to exogenous H_2_O_2_ added directly to the cell culture medium. A classic biological pathway for endogenous H_2_O_2_ production is the cellular binding of lipopolysaccharides (LPS) to macrophage cells^26^,^27^. LPS binds to toll-like receptor 4 (TLR4), which triggers phagocytosis of the LPS/TLR4 complex. Previous studies report that stimulating macrophages with LPS triggers an increase in intracellular H_2_O_2_ levels as a result of the TLR4 signaling pathway^28^,^29^. Murine macrophage cells (RAW 264.7) were incubated with LPS (14.3 μg/mL, *E. coli* J5) at 4 °C, then warmed to 37 °C to initiate LPS signaling. Images were recorded with a spinning disk confocal microscope at a rate of 1 frame per minute over a 2 hour period. In macrophages, filopodia, actin-enriched extensions of the plasma membrane, have been found to be involved with the phagocytic pathway, interacting directly with the target of phagocytosis^30^. Because of this interaction and the spatial orientation of filopodia relative to newly-formed phagosomes, we predicted that the intracellular levels of H_2_O_2_ should be higher near these projections. Images show an elevation in intracellular H_2_O_2_ levels 300 s following the addition of LPS (Fig. 3). This filopodium continues to extend with a concomitant increase in H_2_O_2_ during the period of imaging. By 2040 s, multiple filopodia with a similar increase in localized H_2_O_2_ have formed.

### Spatiotemporal response of cells to H_2_O_2_

**Figure 4:**
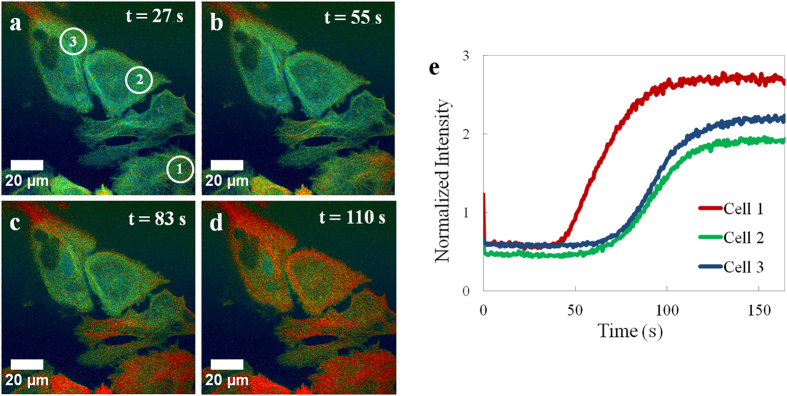
Spatiotemporal effect of H_2_O_2_ on HyPer-Tau expressing HeLa cells. (**a–d**) H_2_O_2_ (200 μM) was added below the cells in the field of view using a microloader pipette tip. Images were recorded at a rate of 1 frame every 0.55 seconds with a spinning disk confocal microscope. The pseudocolor images (0–256 scaling, red is the greatest change) represent the ratio of emission at 516 nm obtained from excitation at 488 nm versus 405 nm. The scale bar is 20 μm. (**e**) Kinetics of oxidation obtained from regions of interest (white circles) within three cells.

Like its commercially available counterparts, HyPer-Tau is highly responsive to H_2_O_2_. Unique to this protein is the spatiotemporal resolution of this response due to its localization at the microtubules. To demonstrate this capability, H_2_O_2_ was added to Hyper-Tau-expressing HeLa cells in a highly localized area using narrow 20 μL microloader pipette tips to deliver the H_2_O_2_ to the cell culture medium. In comparison, for the bolus addition experiments (Fig. 2, Supplementary Figure 3) the entire cell monolayer is exposed to H_2_O_2_ at essentially the same time. In experiments using microloader pipette tips, H_2_O_2_ is added such that one side of the cell culture dish is exposed to H_2_O_2_ before the opposite side is exposed. For example, in Fig. 4, 200 μM H_2_O_2_ was added just below the field of view at t ≈ 10 s. At ~50 s, the cells at the lower edge of the image show the first response to H_2_O_2_. This response migrates from the bottom cells up to the top, reflecting the direction of H_2_O_2_ flow.

## Conclusion

These experiments demonstrate that HyPer-Tau is capable of detecting intracellular H_2_O_2_ in a spatially resolved manner. Cells show a subcellular response to both exogenous (Fig. 2) and endogenous (Fig. 3) H_2_O_2_ with sufficient detail to measure H_2_O_2_ gradients and intracellular kinetics associated with oxidation (Fig. 4). This technical advance will be useful in interpreting redox signaling within the organizational structure that defines cellular morphologies, such as differences between radial diffusion in spherical suspension cells versus polarization gradients across an epithelial cell^31^. A caveat to this tool is the impact on cell morphology which could occur due to Tau overexpression^32^; however, for the transient expression in HeLa and RAW 264.7 cells used in this study, no changes in cell morphology were noted (Supplementary Figure 5). We anticipate HyPer-Tau will provide additional insight into the morphological changes that occur with localized oxidation, such as those occurring at focal adhesions^33^ or in response to endocytosis^34^.

## Materials and Methods

### HyPer-Tau plasmid

The HyPer-Tau plasmid (Supplemenary Figure 1) was generated by conjugating the separate elements coding for HyPer and Tau. The Tau sequence was subcloned from a MAPT plasmid (RC-213312, OriGene, Rockville, MD) via polymerase chain reaction using OneTaq DNA Polymerase (M0480S, New England Biolabs (NEB), Ipswich, MA). The reverse primer inserted a BamHI restriction site with a stop codon in the frame. The PCR product containing the Tau sequence was double digested with BglII (R0144, NEB) and BamHI (R0136, NEB). Tau was cloned into the BglII-BamHI sites of the pHyPer-nuc plasmid (FP944, Evrogen, Moscow, Russia) after excising the nuclear localization signal from pHyPer-Nuc (three copies of the sequence DPKKKRKV). The cloned plasmid was transfected into *E.coli* and the purified DNA was extracted and sequenced.

### Cell culture

HeLa cells (ATCC, Manassas, VA) were maintained in a 37 °C, 5% carbon dioxide environment in Minimum Essential Medium (MEM, 61100–061, Invitrogen, Grand Island, NY) supplemented with 10% (v/v) fetal bovine serum (FBS, 10437–028, Invitrogen). Cells were passaged every 3–4 days, with replacement of the culture medium two days after passage. RAW 264.7 macrophage cells (ATCC) were maintained in a 37 °C, 5% carbon dioxide environment in Dulbecco’s Modified Eagle’s Medium (DMEM, D5796, Sigma-Aldrich, St. Louis, MO) supplemented with 10% (v/v) FBS and 1% (v/v) penicillin-streptomycin (MT-30-001-CI, Mediatech, Manassas, VA). RAW 264.7 cells were passaged every 2–3 days. For fluorescence imaging experiments, cells were cultured in 35 mm glass-bottom dishes and imaged in Leibovitz L-15 medium (21083–027, Invitrogen) or phenol red-free MEM (51200–038, Gibco, Life Technologies).

For fluorescence imaging, HeLa cells were transfected with Fugene-HD (E2311, Promega, Madison, WI) or Lipofectamine 3000 (L3000–015, Life Technologies, Carlsbad, CA). RAW 264.7 cells were transfected using the Neon Transfection System (MPK5000, Life Technologies) and its corresponding reagents (MPK10025, Life Technologies) and then immediately transferred into 35 mm glass-bottom dishes containing complete culture medium.

### Immunofluorescence

At 24 hours post-transfection (Lipofectamine), HeLa cells were washed three times with warm microtubule-stabilizing buffer [80 mM PIPES (P8203, Sigma-Aldrich), 1 mM MgCl_2_, 1 mM EGTA, 4% (w/v) polyethylene glycol (PEG, A162421, Alfa Aeasar, Ward Hill, MA), diluted in water to pH 6.9]^35^. The cells were then fixed and permeabilized with −20 °C methanol for 5 minutes. Cells were incubated with mouse anti-alpha tubulin (α-tubulin) antibody (ab7291, Abcam, Cambridge, MA) at a dilution of 1:200 in HBSS (12025–092, Life Technologies) for 1 hour at room temperature. After the incubation period, the cells were washed with HBSS and incubated with AlexaFluor 647 chicken anti-mouse antibody (A-21463, Life Technologies) at a dilution of 1:1000 in HBSS for 1 hour at room temperature. Before imaging, cells were washed with HBSS. Single antibody controls were used to ensure that no cross-talk occurred between the red and green channel during imaging.

### Fluorescence microscopy

For the majority of experiments (Figs 2, 3, 4), cells were imaged with an inverted microscope (Olympus IX81, Center Valley, PA) equipped with a spinning disk confocal scanner unit (CSU-X1, Yokogawa, Tokyo, Japan), using a 1.42 N.A., 60×, oil immersion objective (Olympus). Figure 1a was imaged with a Zeiss LSM 700 confocal microscope (Jena, Germany). HyPer-Tau was excited separately at 405 nm and 488 nm and the emission collected at 516 nm. Emission was detected with an EMCCD camera (DU-897, Andor). The specific frame rate is provided in the main text. At the start of each experiment, the average emission intensities collected from excitation at 405 nm and 488 nm were set equal by adjusting the laser power, exposure time and gain parameters. For HeLa cells, H_2_O_2_ was added to the existing medium in the optical dish using either regular pipette tips (LTS-20, Mettler-Toledo, Greifensee, Switzerland) before imaging (Fig. 2) or 20 μL microloader tips (930001007, Eppendorf, Hamburg, Germany) during imaging (Fig. 4). Using MetaMorph (Molecular Devices, Sunnyvale, CA) and MatLab-based Biosensor Processing Software 2.1 (Fig. 3)^36^, the ratio of emission from 488 nm to that from 405 nm was calculated at each time point and represented in pseudocolor images.

For Fig. 1b, Structured Illumination Microscopy (SIM) was used to image HyPer-Tau in live cells with sub-diffraction resolution. An Elyra PS.1 microscope (Zeiss, Jena, Germany) with 1.40 N.A., 63×, oil immersion objective was used for imaging. HyPer-Tau was excited at 488 nm and emission was collected at 516 nm. Images were collected at 3 rotations over 5 phases, with a grating period of 28 μm. ZEN 2012 software (Zeiss) was used to produce the final image.

For imaging in response to LPS stimulation (Fig. 3), electroporated RAW 264.7 cells were incubated with 700 μL of cold Leibovitz L-15 medium and 10 μg of *E. coli* J5 LPS (437620-5MG, Calbiochem, EMD Millipore, Billerica, MA) for 15 minutes at 4 °C. After this cold binding, 1 mL of warm medium was added to the cells, and the dish was placed in the heated stage-top incubator for imaging.

## Additional Information

**How to cite this article**: Warren, E. A. K. *et al.* Spatially-resolved intracellular sensing of hydrogen peroxide in living cells. *Sci. Rep.* **5**, 16929; doi: 10.1038/srep16929 (2015).

## Supplementary Material

Supplementary Movie 1

Supplementary Information
