# Supplementary material for: Spatially-resolved intracellular sensing of hydrogen peroxide in living cells
Source: Sci Rep. 2015 Nov 20;5:16929. doi: 10.1038/srep16929 (PMC4653655; doi:10.1038/srep16929)
Supplement: Supplementary Information [file srep16929-s2.pdf]

Supplementary Information for:

Spatially-resolved intracellular sensing of hydrogen peroxide in living cells

Emilie A.K. Warren[1,‡,†], Tatiana S. Netterfield[2,‡], Saheli Sarkar[1,2,3,†], Melissa L. Kemp[2,3]*, and Christine K. Payne[1,3*]

[1]School of Chemistry and Biochemistry, Georgia Institute of Technology, Atlanta, GA 30332, USA. [2]The Wallace H. Coulter Department of Biomedical Engineering, Georgia Institute of Technology and Emory University, Atlanta, GA 30332, USA. [3]Parker H. Petit Institute for Bioengineering and Biosciences, Georgia Institute of Technology, Atlanta, GA 30332, USA. *e-mail: christine.payne@chemistry.gatech.edu; melissa.kemp@bme.gatech.edu

†Present addresses

Ms. Emilie Warren, Baylor College of Medicine, Houston, TX; Dr. Saheli Sarkar, Department of Pharmaceutical Sciences, Northeastern University, Boston, MA

Author Contributions

‡These authors contributed equally.

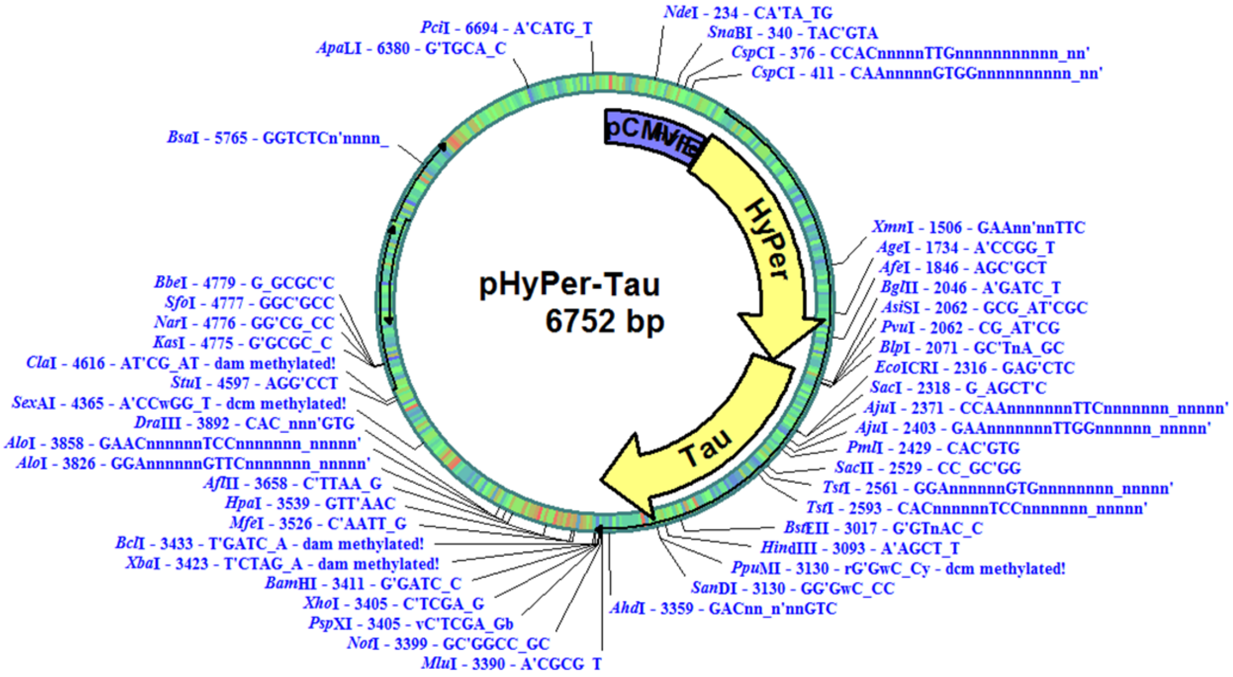

Figure S1. HyPer-Tau plasmid map.

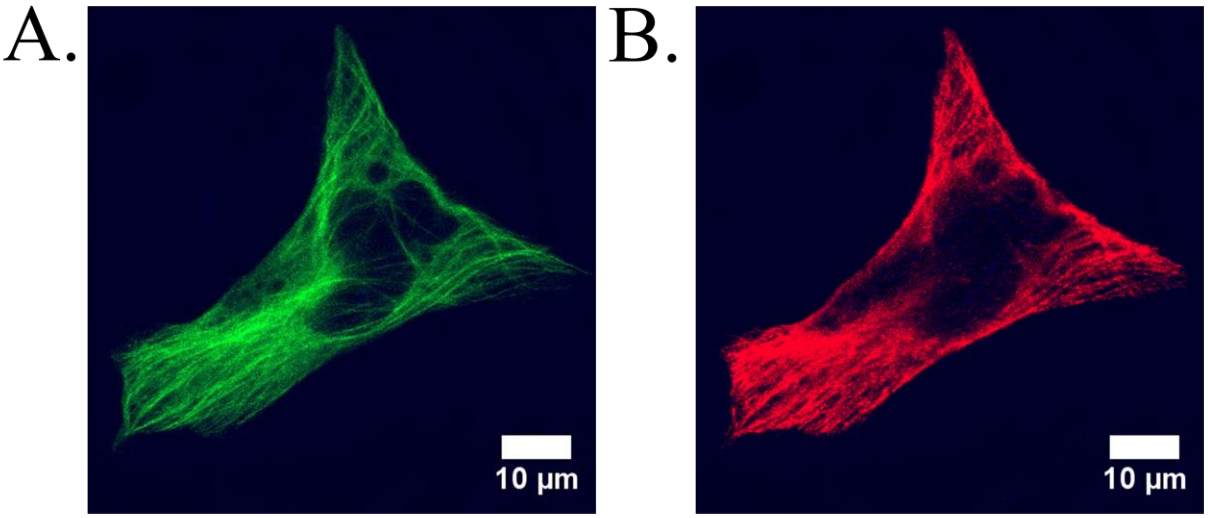

Figure S2. Individual fluorescence microscopy images used to construct Figure 1a. (A) HyPer-Tau (green) transiently expressed in HeLa cells (green). (B) The same cell treated with a primary mouse anti-α tubulin antibody and then labeled with a chicken anti-mouse antibody conjugated to AlexaFluor 647 (red).

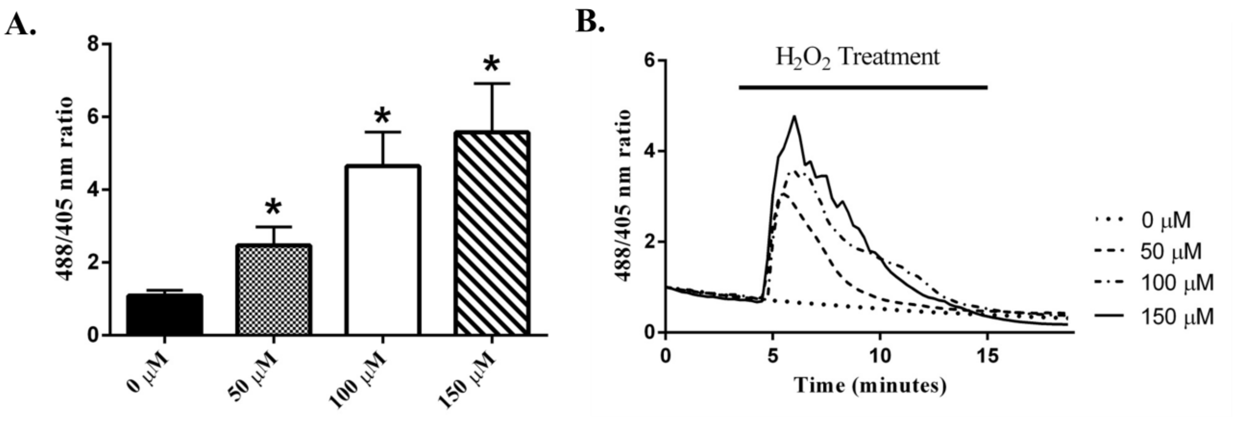

Figure S3. Bolus hydrogen peroxide addition. HeLa cells were imaged before and after treatment with 0, 50, 100, or 150 μM of H_2O_2. Results are represented as both (A) the mean maximum 488/405 nm ratio (n=3 distinct experiments) and (B) the 488/405 nm ratio over time for individual experiments. Error bars represent standard deviation. * indicates a p-value < 0.05 for a two-tailed t-test in comparison to the control with 0 μM H_2O_2. Cells were imaged with a LSM 700 confocal microscope (Zeiss, Jena, Germany) using a 1.40 N.A., 60x, oil immersion objective. Images were recorded every 15 seconds. Five minutes of baseline fluorescence was recorded prior to the addition of H_2O_2. The mean fluorescence intensity at each time point was analyzed post-imaging using the ROI tools on ImageJ (http://rsb.info.nih.gov/ij/).

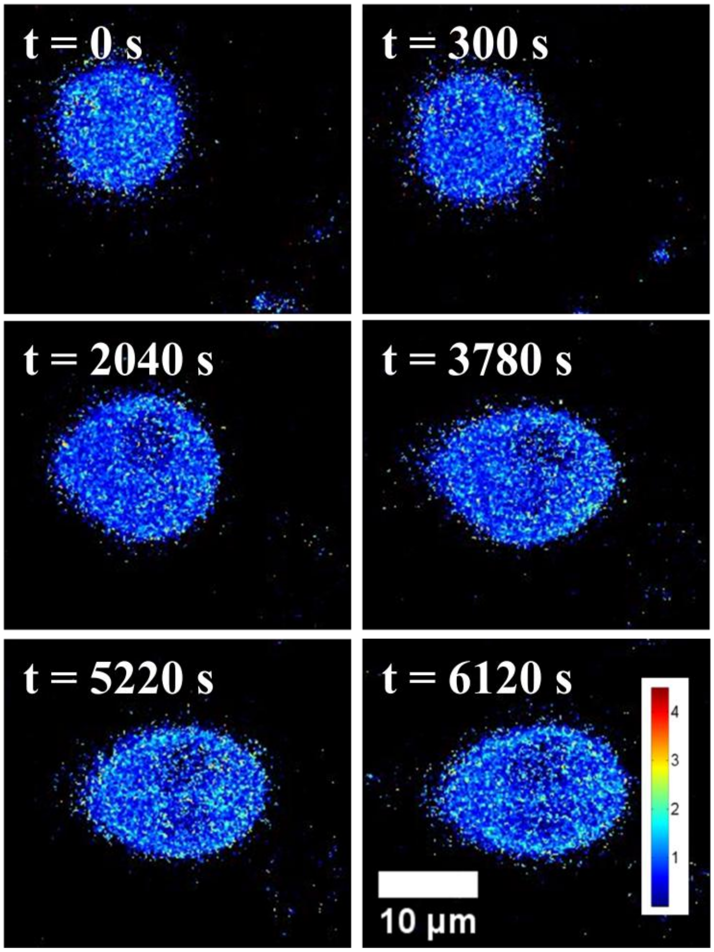

Figure S4. Negative control for Figure 3. Macrophage cell (RAW 264.7) imaged in the absence of LPS. The same conditions were used as described in Figure 3. Images were collected with an inverted microscope (Olympus IX81) equipped with a spinning disk confocal scanner unit (CSU-X1, Yokogawa). Over 2 hours of imaging, no increase in H_2O_2 was observed.

Supplementary Movie 1. A single macrophage cell (RAW 264.7) following stimulation with LPS. Images were collected with a spinning disk confocal microscope (Olympus IX81). An image was recorded every minute for 2 hours at 37°C in Leibovitz's media. A ratio map of the 488/405 nm emission intensity was generated for each frame using the Biosensor Processing Software 2.1 MatLab package.

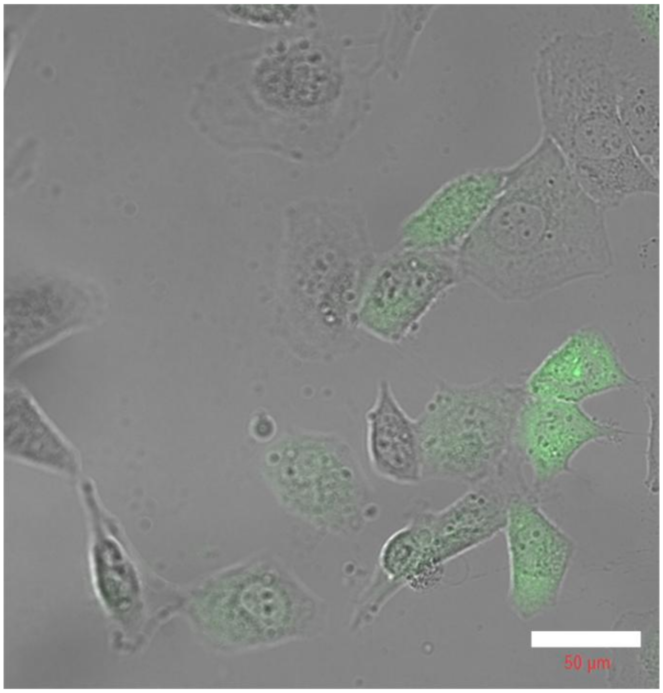

Figure S5. Transiently-transfected HeLa cells imaged at low magnification (Zeiss LSM 700 confocal microscope, 20x objective). The brightfield and fluorescence images are overlaid to compare the morphology of HyPer-Tau expressing cells (green) to those that do not express the fluorescent protein. The scale bar is 50 μm.
